# Acquired cystinuria in a kidney transplant recipient

**DOI:** 10.1093/ckj/sfaf330

**Published:** 2025-11-06

**Authors:** Cécile Martin, Stessy Kutchukian, Laure Ecotière, Héloïse Ducousso, Antoine Thierry, Bertrand Knebelmann, Frank Bridoux

**Affiliations:** Department of Nephrology, Poitiers University Hospital, Poitiers, France; Department of Urology, Poitiers University Hospital, Poitiers, France; Department of Nephrology, Poitiers University Hospital, Poitiers, France; Department of Urology, Poitiers University Hospital, Poitiers, France; Department of Nephrology, Poitiers University Hospital, Poitiers, France; Department of adult Nephrology, APHP-Centre, Necker Hospital, Paris, France; Department of Nephrology, Poitiers University Hospital, Poitiers, France

**Keywords:** cystinuria, genetic disorder, kidney transplant, lithotripsy

## Abstract

Cystinuria is a rare autosomal recessive hereditary disorder characterized by increased urine cystine excretion and recurrent kidney stone formation. Acquired cystinuria after kidney transplantation is extremely rare, with, to our knowledge, a single case described in the literature, albeit without genetic explorations. We herein report a 59-year-old male kidney transplant recipient, without history of urolithiasis, who developed cystine stones in the allograft. Genetic studies revealed a homozygous pathogenic mutation in the *SLC3A1* gene in the deceased donor. This case demonstrates the potential for transmission of cystinuria through the kidney allograft and underlines the value of genetic analysis in the donor.

## INTRODUCTION

Urolithiasis is an uncommon complication after kidney transplantation, affecting 0.4% to 1% of recipients. It may result from various conditions, including altered anatomical features, metabolic disturbances, and, in rare cases, genetic predispositions inherited from the donor [1]. Cystinuria is a rare autosomal recessive disorder affecting *SLC3A1* or *SLC7A9* genes, promoting increased urine cystine excretion favoring crystal precipitation within the distal tubules and subsequent formation of cystine stones [2]. We herein report a case of allograft dysfunction secondary to cystine stones, emphasizing the role of genetic and environmental factors for this rare complication, and the complex management of urolithiasis in kidney allograft recipients.

## CASE PRESENTATION

A 59-year-old male on maintenance hemodialysis for end-stage kidney disease secondary to malignant hypertension, received a first kidney allograft from a deceased donor. His past medical history included hypertension, type II diabetes, dyslipidemia, and subtotal parathyroidectomy for a hyperparathyroidism. The donor was a 44-year-old male without known urolithiasis, but a history of anorexia, depression, daily alcohol consumption, and heavy smoking (40 pack-years). He died from a post-traumatic subdural hemorrhage in the context of alcohol intoxication. The pre-retrieval computed tomography (CT-scan) demonstrated kidneys with normal morphology and no evidence of urinary calculi. Early post-transplantation period was marked by delayed graft function, requiring a single hemodialysis session. Graft function progressively improved afterwards with a serum creatinine level of 0.97 mg/dl at day 60.

**Figure 1:**
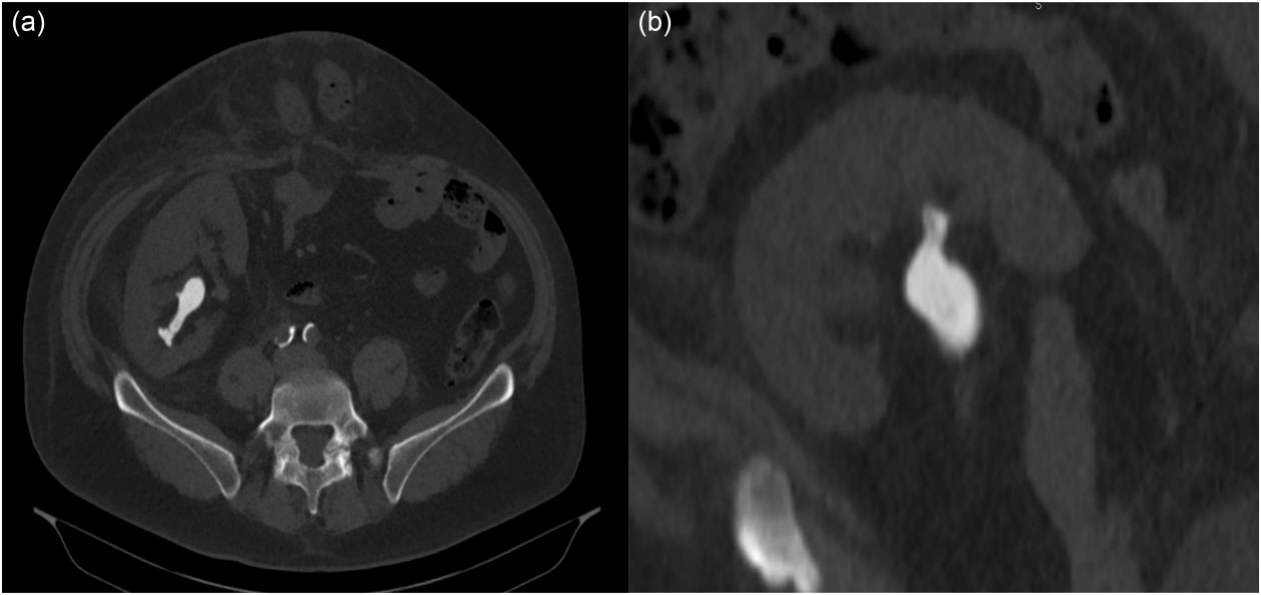
Abdominal NCCT, showing a complex stone of 49 mm in diameter, 690 HU density in the lower calyx of the renal allograft. (**a**) Axial section. (**b**) Coronal section.

At 6 years post-transplantation, a CT-scan performed for an adrenal adenoma fortuitously revealed a complex stone of 49 mm in diameter and 690 Hounsfield unit (HU) density, without pyelocaliceal dilation (Fig. 1). Routine annual kidney ultrasounds were reviewed, without evidence for urolithiasis. A mini-percutaneous nephrolithotomy (PCNL) allowed complete fragmentation and drainage of the calculus. Infrared spectrophotometric analysis confirmed that the stones contained >95% of cystine.

Biologic tests showed serum creatinine 1.01 mg/dl with eGFR (CKD-EPI) of 83 ml/min/1.73 m^2^, calcemia 8.69 mg/dl, calciuria 60.9 mg/24 h (urine calcium/creatinine 0.14 mg/mg), natriuresis 162 mmol/24 h, and urea excretion 17.5 g/24 h. Urine citrate excretion was low (11.5 mg/dl), oxalate excretion was 17.7 mg/l and urine pH was 7.0. Urine cystine/creatinine ratio was 191 mg/mg (normal range 6–34 mg/mg). Crystalluria analysis was unfortunately not feasible.

Genetic analysis performed on the recipient was negative. After approval of local Bioethics Committee and national health authorities, gene panel sequencing conducted on the deceased donor using stored HLA typing samples collected at the time of transplantation, revealed a homozygous pathogenic variant in the *SLC3A1* gene [NM_000341.3: c.1400T>C p.(Met467Thr)], classified as pathogenic, previously reported in individuals affected by cystinuria, confirming the diagnosis of cystinuria transmitted via the kidney allograft [3]. Notably, the recipient of the contralateral kidney did not develop urolithiasis.

The patient followed a high animal-protein diet, with a daily intake exceeding 1.5 g/kg/day. Dietary adjustments were implemented to reduce protein and sodium intake, to increase diuresis, and urine alkalinization with potassium citrate was initiated. Treatment adherence was suboptimal, with a stable urinary pH at 7.0.

Two years after the diagnosis, the recipient experienced acute obstructive pyelonephritis due to calculi migration. No further stone episode occurred. At 9 years post-transplantation, serum creatinine was 1.10 mg/dl, with eGFR of 73 ml/min/1.73 m^2^.

## DISCUSSION

Kidney allograft-transmitted cystinuria is an extremely rare complication, with a single previous reported observation in the literature to our knowledge [4]. Simadri *et al.* described the onset of cystine stones 6 months after kidney transplantation in a 46-year-old male. As in our case, there was no personal or family history of urolithiasis in the donor and the recipient, and the recipient of the contralateral kidney did not experience stone formation in the allograft. Genetic analysis was not performed on the donor.

In the herein described case, the diagnosis of *de novo* cystinuria in the recipient was confirmed by the retrospective identification of a homozygous *SLC3A1* mutation in the donor. This observation confirms that genetic predisposition for cystinuria can be transmitted through organ transplantation.

Medical therapy, including hyperhydration with a target urine output exceeding 3 l per day, low protein diet, and alkalinization aiming for a urinary pH above 7.5 is mandatory for preventing recurrence of cystine stones. Cystine chelators, such as D-penicillamine, may be considered in renal transplant patients with recurrent cystine stones to reduce the risk of severe complications in a single kidney [5]. Moreover, the absence of urolithiasis in the recipient of the contralateral kidney strongly suggests a crucial role for diet in the development of cystine stones. We recommend performing a metabolic workup, including crystalluria analysis and monitoring of urinary pH, in the contralateral recipient.

This case also illustrates the complex management of urolithiasis in kidney transplant recipients. Non-invasive approaches, such as extracorporeal shock wave lithotripsy, are typically avoided in allograft kidneys due to anatomical concerns, leading to reliance on ureteroscopy and PCNL [6]. As repeat PCNL may alter kidney function, ureteroscopy should be considered first.

In conclusion, this case underscores the potential for transmission of cystinuria through the kidney allograft. Careful workup, including metabolic studies, crystalluria and stone composition analysis, is required in kidney transplant recipients with urolithiasis. Diagnosis assessment relies on genetic testing of the donor, that should be considered even in the absence of a known history of urine stones.
